# Design, Development, and Evaluation of Constant Voltage Iontophoresis for the Transungual Delivery of Efinaconazole

**DOI:** 10.3390/pharmaceutics15051422

**Published:** 2023-05-06

**Authors:** Anroop B. Nair, Bandar Aldhubiab, Jigar Shah, Shery Jacob, Mahesh Attimarad, Nagaraja Sreeharsha, Katharigatta N. Venugopala, Alex Joseph, Mohamed A. Morsy

**Affiliations:** 1 Department of Pharmaceutical Sciences, College of Clinical Pharmacy, King Faisal University, Al-Ahsa 31982, Saudi Arabia; 2 Department of Pharmaceutics, Institute of Pharmacy, Nirma University, Ahmedabad 382481, India; 3 Department of Pharmaceutical Sciences, College of Pharmacy, Gulf Medical University, Ajman 4184, United Arab Emirates; 4 Department of Pharmaceutics, Vidya Siri College of Pharmacy, Off Sarjapura Road, Bangalore 560035, India; 5 Department of Biotechnology and Food Technology, Durban University of Technology, Durban 4000, South Africa; 6 Department of Pharmaceutical Chemistry, Manipal College of Pharmaceutical Sciences, Manipal Academy of Higher Education, Manipal 576104, India; 7 Department of Pharmacology, Faculty of Medicine, Minia University, El-Minia 61511, Egypt

**Keywords:** onychomycosis, efinaconazole, transungual, iontophoresis, optimization, antifungal

## Abstract

The efficacy of topical antifungal therapy in onychomycosis has been hindered by the failure of the antimycotic to permeate the nail plate. This research aims to design and develop a transungual system for the effective delivery of efinaconazole utilizing constant voltage iontophoresis. Seven prototype drug-loaded hydrogel formulations (E1–E7) were prepared to assess the influence of solvent (ethanol) and cosolvent (Labrasol^®^) on transungual delivery. Optimization was performed to evaluate the effect of three independent variables; voltage, solvent-to-cosolvent ratio, and penetration enhancer (PEG 400) concentration on critical quality attributes (CQAs), such as drug permeation and loading into the nail. The selected hydrogel product was characterized for pharmaceutical properties, efinaconazole release from the nail, and antifungal activity. Preliminary data indicates ethanol, Labrasol^®^, and voltage influence the transungual delivery of efinaconazole. Optimization design indicates a significant impact by applied voltage (*p*-0.0001) and enhancer concentration (*p*-0.0004) on the CQAs. Excellent correlation between selected independent variables and CQAs was confirmed by the high desirability value (0.9427). A significant (*p* < 0.0001) enhancement in the permeation (~78.59 µg/cm^2^) and drug loading (3.24 µg/mg) was noticed in the optimized transungual delivery with 10.5 V. FTIR spectral data indicates no interaction between the drug and excipients, while the DSC thermograms confirmed the amorphous state of the drug in the formulation. Iontophoresis produces a drug depot in the nail that releases above the minimum inhibitory concentration level for an extended period, potentially reducing the need for frequent topical treatment. Antifungal studies further substantiate the release data and have shown remarkable inhibition of *Trichophyton mentagrophyte*. Overall, the promising results obtained here demonstrate the prospective of this non-invasive method for the effective transungual delivery of efinaconazole, which could improve the treatment of onychomycosis.

## 1. Introduction

Onychomycosis is a chronic, progressive fungal disease that typically damages the toenail unit resulting in hardening, yellowing, breaking, detachment from the nail bed, and paronychia [1]. It is typically infected by dermatophytes such as *Trichophyton*, *Microsporum*, or *Epidermophyton genera*; nevertheless, non-dermatophytic molds and yeasts may also be implicated. Onychomycoses are more difficult to treat than most dermatophytosis because of intrinsic factors of the nail, such as the hardness, concealment of pathogens within the keratin layers, and gradual growth of the nail. The selection and effectiveness of antifungal therapy are based on the nature of onychomycosis, the number of distressed nails, and the severity of symptoms. Four different types of onychomycosis are described depending on clinical presentation, location, and pattern of fungal infection [2]. Underlying conditions, such as tinea pedis, malignancy, age, obesity, and comorbidities like diabetes, peripheral arterial disease, and psoriasis, can increase the susceptibility to the disease [3]. If left untreated, this disease will cause thickness of the nail, onycholysis, secondary bacterial infection, and, thus, disrupting physical, emotional, and social activities [4]. An important formulation challenge during the treatment of onychopathies is to maintain a therapeutically effective drug concentration higher than the minimum inhibitory concentration (MIC) corresponding to the infected microorganism in the nail layers [5,6]. Presently, both oral and topical therapies are used as standard treatment strategies for the clinical management of onychomycosis and treatment-resistant dermatophytosis [7,8]. The key disadvantages of oral antifungal therapy are a lower cure rate, the development of resistant strains, serious systemic toxicity, and interactions with other drugs [9,10]. On the other hand, topical applications possess several advantages, including target drug delivery and enhanced patient acceptability [9]. Ungual and transungual drug delivery has gained much attention in the last two decades, mainly because of the need for a safe and effective drug therapy that can surmount the issues with oral administration [6]. Providing the drug directly into and near the infected nails can avoid the risk of systemic exposure. Currently, different topical drug products are available for nail treatment but without much clinical success [11]. The low efficacy in topical therapy is primarily due to the hindrance caused by reduced permeation of drugs via the hard, impermeable, complex, multilayered structure of keratinized nail plate [12,13]. In this context, an efficacious topical therapy that can provide the clinical and mycological cure for onychomycosis is the need of the hour.

Different classes of chemicals like keratolytics, inorganic salts, glycols, reducing agents, and oxidizing substances were evaluated for their prospective to improve the trans-nail drug transport [14,15,16,17]. Many of these agents have been found to be only moderately efficient and have not been able to effectively increase drug permeation. Few investigations have evaluated the feasibility of combination approaches to improve the transungual delivery of various antifungal agents [18,19]. Alternatively, nanovesicular drug carriers, namely liposomes, ethosomes, nanoemulsions as well as microneedle procedures, were assessed for their potential transungual drug permeation [20,21,22,23]. Various physical approaches, such as iontophoresis, laser technique, microporation, ablation, abrasion, and ultrasound, were also evaluated to check their prospective to enhance the drug into and through the nail [24,25,26,27,28]. Among these, iontophoresis remains a viable method for delivering charged molecules or nanocarriers [29,30,31]. Indeed, the feasibility of constant current iontophoresis in nail therapy was demonstrated in various studies [4,32,33]. One of the major concerns noticed with ungual iontophoresis is the ramping of voltage during the treatment as a consequence of the high resistance produced by the nail plate. Alternatively, constant voltage iontophoresis could be more patient-friendly and can enhance drug permeation through the nail membrane [34].

**Figure 1:**
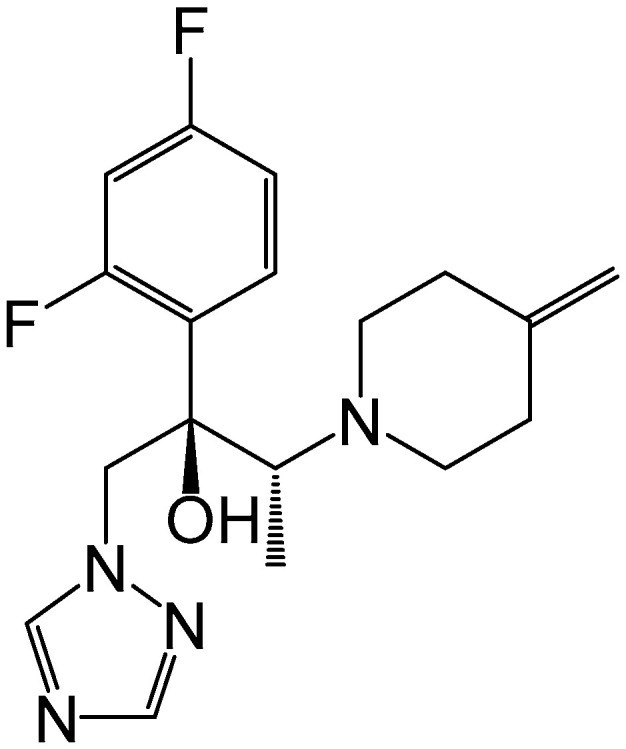
Molecular structure of efinaconazole.

Efinaconazole is an azole antifungal agent belong to 1,2,4-triazol chemical class (Figure 1). The drug molecule’s heterocyclic nature is basic and tends to exist in hydrochloride salt form with hydrochloric acid. This salt formation could be confirmed by the complete solubility of efinaconazole in the acidic pH. In general, an ionized form of efinaconazole in polar solvents will have a positive charge. The compound exhibits predicted cLogP and pKa of 2.1452 and 12.11, respectively. Efinaconazole obeys the Lipinski rule, where the molecular mass is 348 Da, and the number of hydrogen bond donors and acceptors is 1 and 6, respectively. All the above-mentioned physicochemical characteristics suggest that efinaconazole in acidic pH is suitable for anodal iontophoretic delivery. Efinaconazole demonstrated a potent broad spectrum of activity against various *Trichophyton* species as well as *Candida albicans* in comparison to presently available antifungals that are used in onychomycosis [35]. In addition, efinaconazole exhibits significant inhibition of molds and yeasts, while it is a choice in case of resistance or ineffective treatment of onychomycosis by terbinafine [36]. The antifungal susceptibility test suggests the antimycotic effect of efinaconazole against *T. rubrum* (MIC: 0.039 μg/mL) and *T. mentagrophyte* (MIC: 0.044 μg/mL) was better in comparison to terbinafine after 10 days [37]. Moreover, MIC levels demonstrated by efinaconazole indicated lower levels of resistance and better therapeutic efficacy compared to terbinafine. Undoubtedly, efinaconazole showed relatively higher susceptibility against *C. albicans* with a low MIC (0.001 μg/mL) than itraconazole (0.015 μg/mL). Various attempts have been made to deliver efinaconazole through the nail by formulating it into solution [38,39,40], lacquer [41], and spanlastic nanovesicles [42]. However, no physical enhancement approaches have been attempted to deliver efinaconazole through the nail. Thus, this study aimed to develop a transungual iontophoretic delivery system of efinaconazole by employing a constant low-voltage iontophoretic approach. The best formulation designs were produced using a custom design that matched the given experimental conditions. The three independent variables selected were voltage range, the solvent-to-cosolvent ratio, and enhancer concentration, while the critical quality attributes (CQAs) for the design were determined to be the drug penetration and accumulation in the nail. The selected hydrogel product was subsequently evaluated for in vitro parameters and additionally for antifungal efficacy.

## 2. Materials and Methods

### 2.1. Chemicals

Efinaconazole was received as a gratis sample kindly donated by Lupin, Pune, India. Butylated hydroxytoluene (BHT), disodium ethylenediaminetetraacetic acid (EDTA), isopropyl myristate, isopropyl palmitate, Labrafil^®^, Labrasol^®^, *N*-Methyl-2-pyrrolidone, polyethylene glycol 400 (PEG 400), polyvinylpyrrolidone K30 (PVP K30), propylene glycol, Transcutol^®^ P, and Tween 80, were obtained commercially (Sigma Aldrich, St. Louis, MO, USA). Ethyl alcohol, isopropyl alcohol, acetonitrile, and potassium dihydrogen phosphate were obtained from Qualikems, New Delhi, India.

### 2.2. Determination of Efinaconazole by Reverse Phase-High-Performance Liquid Chromatography (RP-HPLC)

Efinaconazole was quantified by RP HPLC (Agilent Technologies, Waldbronn, Germany) method using an Agilent Eclipse Plus C_18_ column to facilitate chromatographic separation. Efinaconazole was eluted with acetonitrile and potassium dihydrogen phosphate (20 mM) in a ratio of 70:30 (*v*/*v*), and the volume of samples injected was 20 µL. The eluent was allowed to flow at a velocity of 1.2 mL/min at room temperature, and the eluent was monitored at 210 nm using ChemStation software (Agilent Technologies, Waldbronn, Germany).

### 2.3. Drug Solubility in Solubilizers

The saturation solubility of efinaconazole was assessed for the selected solubilizers (ethyl alcohol, isopropyl alcohol, Labrasol^®^, Labrafil^®^, propylene glycol) at room temperature (25 ± 1 °C) by the equilibrium solubility method described in the literature [43]. A surplus amount of the drug was added in different solubilizers (1 mL), taken in separate vials and shaken for 24 h in a horizontal shaker. The suspensions were centrifuged for 10 min at 4000 rpm and further filtered using a 0.22 µm membrane filter unit. The samples were further diluted and estimated for efinaconazole by HPLC.

### 2.4. Screening of Permeation Enhancers

Screening of chemical enhancers was assessed by measuring the amount of efinaconazole diffused into the nail plates as described elsewhere [16]. Briefly, a drug solution (10 mg/mL) containing various enhancers (20% *w*/*w*, isopropyl myristate, isopropyl palmitate, *N*-methyl-2-pyrrolidone, PEG 400, and Transcutol^®^ P) were prepared. Human nail clippings (~2 mm diameter) were weighed and placed in a glass vial, and 2 mL of enhancer solution was added and kept for 24 h. The nail pieces were taken out and cleaned with alcohol, and the surface was wiped. The procedure that was described earlier was used to extract efinaconazole from nail samples [39]. In brief, the nail pieces were digested in NaOH solution (1 M, 1.5 mL) by placing them at 60 °C for 4 h and then combined with 1 mL of methanol and ethyl alcohol (1:1). The efinaconazole solution was then centrifuged at 4000 rpm for 10 min, and the transparent supernatant was collected and dried. The residue was mixed in 10 mL containing 1:1 ethyl alcohol and mobile phase and analyzed by HPLC.

### 2.5. Preparation of Primary Formulations

Table 1 summarizes the embodiments used in the preparation of primary formulations (E1–E7) with different ratios of solvent and cosolvent (1:1, 2:1, 3:1, 4:1, 3:2, 5:2, and 5:3). Hydrogels were formulated by weighing the required quantities of ingredients mentioned in Table 1. Briefly, EDTA, BHT, Tween 80, and PEG 400 were added to water by continuously stirring and heating at 45 °C. The clear solution was cooled, and added efinaconazole, which was previously dissolved in ethyl alcohol and Labrasol^®^. In the end, PVP K30 was added with continuous stirring, the formulation pH was adjusted to 3 using 1 M hydrochloric acid, and water was added to make up the final volume.

### 2.6. Permeation and Drug Loading in Nail

For in vitro passive permeation, nail clippings from healthy participants were used. Nail plates were soaked in saline solution for 1 h and then mounted on a nail holder. The nail-containing adapter was kept between the upper and lower chamber of a typical diffusion setup (Logan Instruments Ltd., Somerset, NJ, USA). The drug diffusion area of the nail was 0.2 cm^2^. Each formulation (E1–E7, 500 μL) containing an equivalent amount of drug (37.5 mg) was applied to the upper chamber, and permeation was continued for 6 h. The sink condition was maintained by adding 0.2% *w*/*v* of sodium lauryl sulfate, as reported in the literature [40]. It was suggested that the solubility of efinaconazole increased substantially in the presence of sodium lauryl sulfate above critical concentration, potentially due to the formation of micelle in aqueous media [40].

Passive permeation data indicated the highest efinaconazole penetration and drug loading in the nail with formulation E1; hence, this was selected and further evaluated for the effect of iontophoresis. The influence on efinaconazole penetration through the nail membrane and loading inside the nail on the selected formulation was tested at two applied voltages (6 V and 12 V) for 6 h using two batches E1a (6 V) and E1b (12 V).

Iontophoresis permeation experiments were carried out similarly to that of passive permeation. Anodal iontophoresis was carried out with Ag–AgCl electrodes (Alfa Aesar, Wardhill, MA, USA), which were placed in upper and lower chambers. Different voltages were applied using a custom-made direct current electrical supply (Rolex Scientific Engineers, Ambala, India) and were checked with an electronic meter (Mextech Mas830L, Mextech Technologies, Mumbai, India). The nail was removed after the experiments, and the surface drug was cleaned with 1 mL of ethyl alcohol five times. The exposed area of the nail plate was cut, weighed, and used for assessing drug content. The extraction of efinaconazole from nail samples was done as described in Section 2.4.

### 2.7. Characterization of Preliminary Formulations

The transparency of the formulations (E1–E7) was assessed by physically looking for any particles, precipitation, or turbidity. An electronic pH meter (Mettler Toledo MP-220, Greifensee, Switzerland) was used to measure the pH of the formulation at 25 ± 1 °C. Drug loading was determined by weighing 1 g of hydrogels and placed in separate glass vials. To this added 5 mL of HPLC mobile phase and stirred to dissolve the drug. The solution was then filtered and analyzed by HPLC. The percentage drug loading and loading efficacy were computed by the equations described elsewhere [34]. The viscosity of the prepared formulation (E1–E7) was determined with the S18 spindle at 50 rpm utilizing a viscometer (Brookfield, Middleborough, MA, USA) at 25 °C.

### 2.8. Custom Design

The custom design feature of JMP^®^ makes it possible to create smart designs more quickly, with less effort, and with better resource management for running experiments. A proprietary design platform employing JMP^®^ version 13.2 software (JMP Statistical Discovery, Cary, NC, USA) was used to create the best formulation designs specifically tailored for the current experimental conditions. The independent variables used for the custom design are the voltage range, the solvent-to-cosolvent ratio, and the concentration of the enhancer (PEG 400). The penetration of the drug through the nail and the drug loading into the nail was chosen as the critical quality attributes (CQAs) for the design.

#### Data Analysis and Interpretation of Results

The various formulation batches were created in accordance with the precise specifications. The CQAs were examined in the prepared formulations. To verify the model fit, the collected data was incorporated into the design. The trial model that matches the experimental design (DoE) data was predetermined and was consistent with the experimental purpose and data collection strategy. Multiple regression modeling was used to statistically examine the data that was gathered. Statistical significance is attained when a variable’s *p*-value is less than 0.05.

### 2.9. Evaluation of Hydrogels

#### 2.9.1. Conductivity

The conductivity of formulation batches (F1–F14) and drug solution (3.5% *w*/*w*, pH 3) were measured at room temperature (25 ± 1 °C) using a conductivity meter (Analab Scientific Instruments, Vadodara, India) with a selected cell constant value of 1.

#### 2.9.2. FTIR Spectroscopy

The spectral characteristics of the efinaconazole, placebo, and optimized hydrogel were measured with a Jasco spectrometer (Tokyo, Japan). Chloroform (0.1 mL) was used to dilute the liquid samples before placing them into the device. Each sample underwent at least 32 scans while the resolution was set at 16 cm^−1,^ and the spectra were recorded between 400 and 4000 cm^−1^.

#### 2.9.3. DSC Analysis

A Hitachi DSC instrument (Tokyo, Japan) was used to measure the thermogram of efinaconazole, physical mixture (efinaconazole and PVP K30), and optimized formulation. The drug and physical mixture were used as such, while the freeze-dried samples of hydrogels were used for analysis. The sample (5 mg) was fixed in an aluminum pan, whereas a blank pan was used as a control [44]. Samples were heated from 30 °C to 350 °C at a rate of 10 °C/min. Thermograms were recorded in an inert atmosphere by purging nitrogen gas at a flow rate of 30 mL/min.

#### 2.9.4. Field Emission Scanning Electron Microscopy (SEM)

The microstructure of the hydrogel was checked using an SEM (Ultra-55, Carl Zeiss, Oberkochen, Germany). Similar to this, the topography of normal human nail plate (control), nails treated with formulation (passive), and nails treated with formulation and iontophoresis (10.5 V) were captured at 1000× magnification. The SEM images were obtained in low vacuum mode operation at an acceleration voltage of 5 kV.

### 2.10. Release of Efinaconazole from Loaded Nails

To assess the drug release, the nail plates were first loaded with optimized formulation and applied constant voltage iontophoresis (10.5 V) or passive (0 V) as described in Section 2.6. The protocol was fixed to mimic the in vivo conditions; therefore, the nail surface was cleaned while keeping the nail inside the nail adapter. The receiver chamber was refilled with fresh media, and samples (1 mL) were obtained for seven days to measure the release of efinaconazole.

### 2.11. Antifungal Study

The preparation of SDA (Sabouraud Dextrose Agar) plates followed the manufacturer’s recommendations. In brief, sterilized SDA was poured into a single 90 mm × 15 mm petri dish and allowed for solidification at room temperature. A slurry of Trichophyton mentagrophyte (ATCC 24953) was grown on a PDA medium and kept at ~28 °C for ten days [45]. The applicator was dipped in a slurry of fungus (1.0 × 10^6^ CFU/mL) and applied on the surface of the plate to inoculate SDA plates. The drug loading to the nail was done as described in Section 2.6. The appropriate area of the nail was cut, and the nail’s lower surface was placed over the SDA plates. The zone of inhibition (mm) was determined after 4 days of incubation at 30 °C [46].

### 2.12. Formulation Stability

The selected formulation was placed in glass tubes and kept in a desiccator for three months. The product was stored at 25 °C and 60% RH [47] and assessed for color change, homogeneity, aggregation, sedimentation, drug loading, pH, and viscosity.

### 2.13. Statistical Evaluation

The number of trials in each experiment is six, and the data displayed are mean ± SD. GraphPad Prism software (version 6, GraphPad, San Diego, CA, USA) was used for meaningful comparisons using the *t*-test or ANOVA. The differences between values that produced a *p*-value less than 0.05 were regarded as statistically significant.

## 3. Results and Discussion

An HPLC method was established for the quantification of efinaconazole in samples. The drug was eluted at 2.27 ± 0.04 min (Figure S1) and showed a good calibration curve in the concentration range of 5 ng/mL–500 ng/mL with an excellent regression coefficient (r^2^ = 0.9998). The linear equation generated from the calibration curve was used for the quantification of efinaconazole from sample solutions. Following the system suitability test, the optimized technique for efinaconazole was validated by ICH recommendations and showed a good LOD (1.07 ng/mL) and a LOQ (3.32 ng/mL). This analytical approach also demonstrated good accuracy, which was supported by good percentage recovery and low percentage relative error. The precision of the HPLC technique was further supported by the low relative standard deviation for intra-day (1.62%) and inter-day (1.45%), respectively.

In general, both drug and formulation composition influence the efficacy of iontophoretic transport of drugs across biological membranes [48,49]. Therefore designing a formulation wherein the drug particle is charged is of utmost importance. On the other hand, water-based formulations are generally used for the iontophoretic delivery of drugs [17,24,50]. Hence, a hydrogel composition suitable for the iontophoretic delivery of efinaconazole was designed and developed.

### 3.1. Selection of Solubilizer and Permeation Enhancer

**Figure 2:**
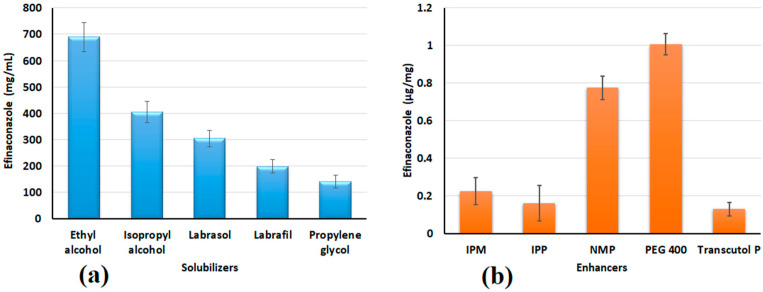
Screening of solubilizers/enhancers to determine the (**a**) solubility of efinaconazole and (**b**) permeation from various enhancers. The value represented is the mean ± SD (n = 6). IPM—isopropyl myristate, IPP—isopropyl palmitate, NMP—n-methyl-2-pyrrolidone, PEG 400—polyethylene glycol 400.

Typically, solubility is considered an important physicochemical property during drug design and drug product development. Developing an aqueous-based formulation of efinaconazole is challenging because the measured drug solubility indicates it is insoluble in water (0.003 mg/mL). The initial phase of the study was performed to assess the solubility of efinaconazole by mixing it with different alcohols or nonionic surfactants, which are generally used as solubilizers in topical formulations. The measured efinaconazole solubility in tested solvents is represented in Figure 2a. In the current experimental conditions, the efinaconazole solubility decreases in the following order, ethanol > isopropyl alcohol > Labrasol^®^ > Labrafil^®^ > propylene glycol. Based on the results, ethanol was selected as the primary solvent as the solubility was high (689.63 ± 55.16 mg/mL). Among the nonionic water-dispersible surfactants tested, Labrasol^®^ showed greater solubility when compared with a similar class of surfactant, Labrafil^®^, hence Labrasol^®^ was selected as a co-solvent.

The selection of appropriate penetration enhancers is critical in order to promote drug diffusion into biological barriers. The combination of chemical enhancers and iontophoresis also yielded synergetic drug transport [24,51]. Five well-known permeation enhancers were selected as these chemical agents have demonstrated their potential to enhance nail drug permeation in various studies [24,52]. The amount of efinaconazole loaded into the nail with various enhancers tested is presented in Figure 2b. It is apparent from the figure that among the five permeation enhancers tested, PEG 400 has shown greater drug loading followed by *N*-Methyl-2-pyrrolidone > Isopropyl myristate > Isopropyl palmitate > Transcutol^®^ P. As the efinaconazole nail permeation was higher with PEG 400, it was included in the formulation. It is described in the literature that low molecular weight PEGs have the ability to increase water intake in nails, which finally becomes soft and reduces endurance, and increases permeability [53]. Indeed, the increased water absorption in the nail causes the keratin to be more hydrated, which improves the diffusion of drugs.

### 3.2. Preparation of Primary Formulations

Seven prototype formulations (E1–E7) were prepared by incorporating various pharmaceutically acceptable excipients mentioned in Table 1. The developed hydrogels have solvent (ethanol), cosolvent (Labrasol^®^), surfactant (Tween 80), permeation enhancer (PEG 400), viscosity modifier (PVP K30), and stabilizers (EDTA, BHT). Different formulation batches (E1–E7) were developed by changing the ratios between solvent and co-solvent (1:1, 2:1, 3:1, 4:1, 3:2, 5:2, and 5:3) while the amounts of the other ingredients was kept constant. In the proposed topical formulation, ethanol was used as a primary solvent to dissolve efinaconazole. It was disclosed that lipophilic enhancers can enhance the nail permeation of efinaconazole [40]. Labrasol^®^ (caprylocaproyl Polyoxyl-8 glycerides) is an oily non-ionic surfactant, which is extensively used in skin and nail formulations as a solvent and permeation enhancer [54,55,56]. The solubility of efinaconazole in Labrasol^®^ is relatively high (>300 mg/mL), and hence this surfactant is preferably used in topical solutions of efinaconazole to keep the drug dissolved when ethanol evaporates, which in turn can improve drug penetration. Hence, Labrasol^®^ was selected and included in the formulation. Studies with efinaconazole demonstrated that transungual formulation developed with low surface tension could provide effective drug penetration into inaccessible crevices and air pockets typically exist in nail beds of onychomycosis [35]. Hence, Tween 80 was used as the surface-active agent to decrease the surface tension of the current formulation. Designing topical transungual preparations with sufficient permeation of the drug through the hard, tightly packed keratinized layers, including the nail plate, is one the key factors that determine their therapeutic efficacy. Low molecular weight PEGs are capable of augmenting the ungual permeation of drugs due to their hydration effect, as described before. As PEG 400 showed higher efinaconazole permeation in the screening study, it was incorporated into the formulation. PVP has been demonstrated as a highly adaptable multifunctional material with high aqueous solubility, biocompatibility, and stability and is widely used in tissue engineering and drug delivery [57]. Due to the hydrogen bonding or complexation effect, the viscosity of PVP is increased, and this further inhibits the formation and growth of crystallized nuclei, hence keeping the drug in amorphous form. In addition, the viscosity of PVP solution is generally not influenced by the pH of a formulation [58]. Furthermore, PVP can also increase the solubility and dissolution rate of the poorly soluble drug. Considering all these advantages, PVP was selected as a viscosity builder in the proposed formulation. It has been reported that efinaconazole preparation undergoes oxidative reactions [39,40], therefore antioxidants like BHT and EDTA were included in the current formulation.

### 3.3. Permeation and Drug Loading in Nail

**Figure 3:**
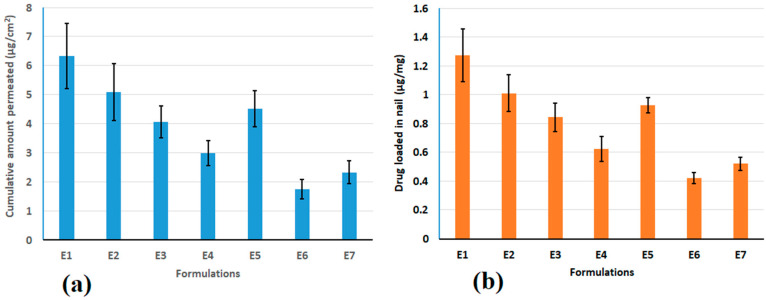
Amount of efinaconazole (**a**) diffused through the nail plate and (**b**) loaded in nails from primary formulations at 6 h. The value represented is the mean ± SD (n = 6).

Table 2 summarizes the results of the characterization of preliminary preparations (E1–E7). The pH and % drug loading did not significantly differ between developed formulations. A slight increase in viscosity occurred with batches E5 < E6 < E7 possibly because of an increase in the quantity of Labrasol^®^ in the respective formulations. Figure 3 presents the cumulative amount of efinaconazole diffused across the nail plate and loaded in nails from primary formulations. As shown in Figure 3, the amount of efinaconazole permeated (6.32 ± 1.12 µg/cm^2^) and drug deposited in the nail (1.27 ± 0.18 µg/mg) were found high in batch E1 and was found to decrease in other batches as E2 > E5 >E3 > E4 > E7 > E6.

The data reveal that the formulations (E2–E7) with an increase in ethanol amount showed reduced transungual permeability as well as nail drug deposition when compared to formulation (E1) with low alcohol content (5%). On the other hand, incorporating a similar percentage of ethyl alcohol (15%) demonstrated a comparable amount of efinaconazole diffusion and deposition in the membrane (E3 and E5). Several variables, especially nail hydration and swelling, affect the permeability of the nail plate [17,59]. It has been described that a hydrated nail showed greater drug permeation, indicating that the nail plate functions like a hydrogel. In contrast, the alcohols and esters are prone to reduce nail hydration and swelling, which in turn cause higher resistance of the ungual barrier [60,61]. A slight increase in drug permeation and drug deposition was noticed with E5 might be due to the inclusion of higher Labrasol^®^ (10%) compared to E3 (5%). It has been demonstrated that Labrasol^®^ has the potential to augment the permeation of various drugs across nails [54,55,56,62], and other biological barriers like GIT [63], cornea [64], and the skin membrane [65]. As the drug diffusion and accumulation in the nail were higher in E1, this formulation was elected as the best-suited hydrogel and employed in additional iontophoresis investigations. Two alternative voltages—batch E1a (6 V) and E1b (12 V) were examined for permeation of 6 h. The findings demonstrate that batch E1b (87.82 ± 6.23 µg/cm^2^) had superior diffusion (*p* < 0.0001) than batch E1a (38.70 ± 3.89 µg/cm^2^), which was 6 and 14 times over E1, respectively. Indeed, the data here demonstrated that the increase in voltage substantially influences the efinaconazole permeation through the nail. A similar observation was also noticed with drug loading in the nail, wherein a significantly (*p* < 0.005) higher amount was accumulated in E1b (3.54 ± 0.39 µg/mg) than in E1a (2.06 ± 0.30 µg/mg). In general, the data here indicates ethanol (solvent), Labrasol^®^ (cosolvent), and voltage influence the transungual delivery of efinaconazole.

### 3.4. Custom Design

Voltage (4.5, 7.5, and 10.5 V), the solvent to cosolvent ratios (1:0.5, 1:1, and 1.5:1), and enhancer concentration (20, 25, and 30% *w*/*w*) were selected as independent variables and investigated in 3 categorical levels to assess the CQAs (the permeation and drug loading). The application time was fixed at 6 h. Based on the initial assessment of variables, the algorithm has found a suitable design with 15 default experimental runs (Table 3). 

#### 3.4.1. Design Verification by Color Map and Design Diagnostic

1. Color Map

**Figure 4:**
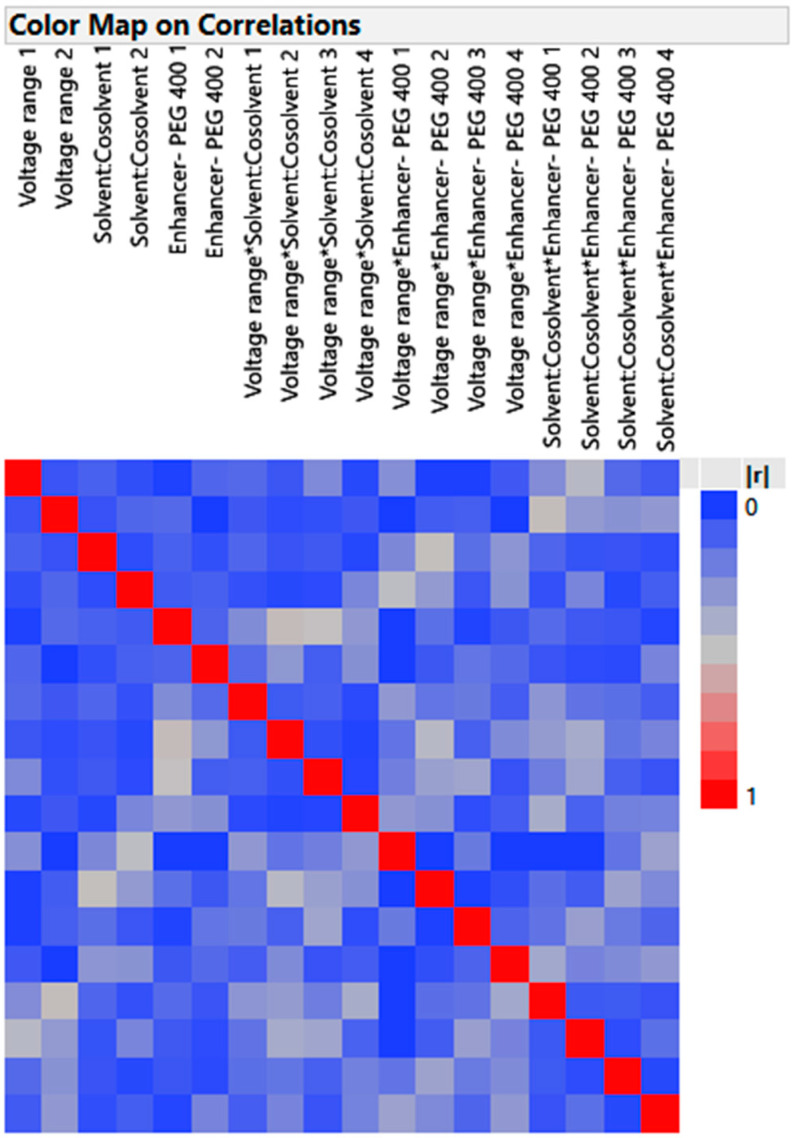
The color map on correlation.

The effect of each independent variable on the responses (penetration of the drug through the nail and drug loading into the nail) was estimated using the color map (Figure 4) alone or in combination with other variables. While the deep red, brown, and blue colors are ranked in declining order of efficacy in eliciting the desired responses, the bright red regions are the most striking combination. The primary effects’ correlations with other effects are shown in deep blue, and zero correlations are indicated by shade. When the major effects are orthogonal and have a value of zero, they can all be independently calculated. The primary diagonal in the color map is occupied by the color red. Each term is perfectly associated with itself, as seen by the red color, which displays the absolute correlation of one. As a result, no two-way interactions totally obscure any principal impacts. Therefore, the color map shows that the design is perfect for factor screening so that efinaconazole delivery can be made that meets all of the quality requirements that have already been set.

2. Design Diagnostics

The design diagnostics provide the design efficiency, which is expressed as ratios or percentages that correspond to the efficiency of the theoretically suitable design. The D-efficiency (96.79), G-efficiency (82.52), and A-efficiency (93.33), all of which are high for the custom design, show that it is the best choice for the main effects model in all three ways. 

#### 3.4.2. Actual vs. Predicted Plots

**Figure 5:**
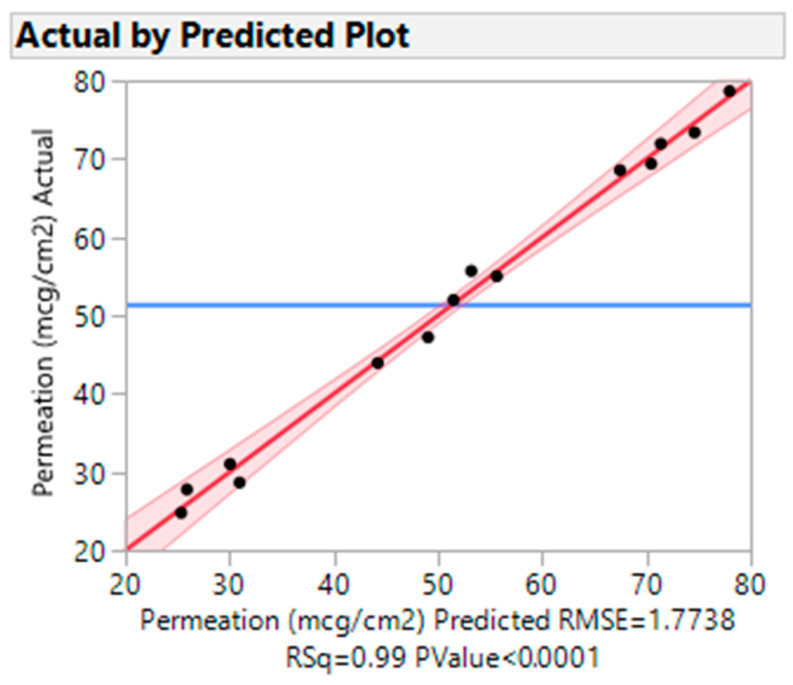
Actual vs. predicted plot for drug permeation.

The experimental vs. predicted graphs are used to generate the analysis’s overall summary. The graphic compares the actual response values to those predicted by the model. The diagonal line is the anticipated mean, and the horizontal line is the overall mean. The bands represent the anticipated means of confidence bands. The graphic (Figure 5) shows the obtained experimental vs. predicted charts for drug permeation. The statistical significance was shown by the fact that the drug permeation had a *p*-value of 0.0001 and an R-square value of 0.99. The scaled estimates for drug permeation through the nail are presented in Figure S2.

**Figure 6:**
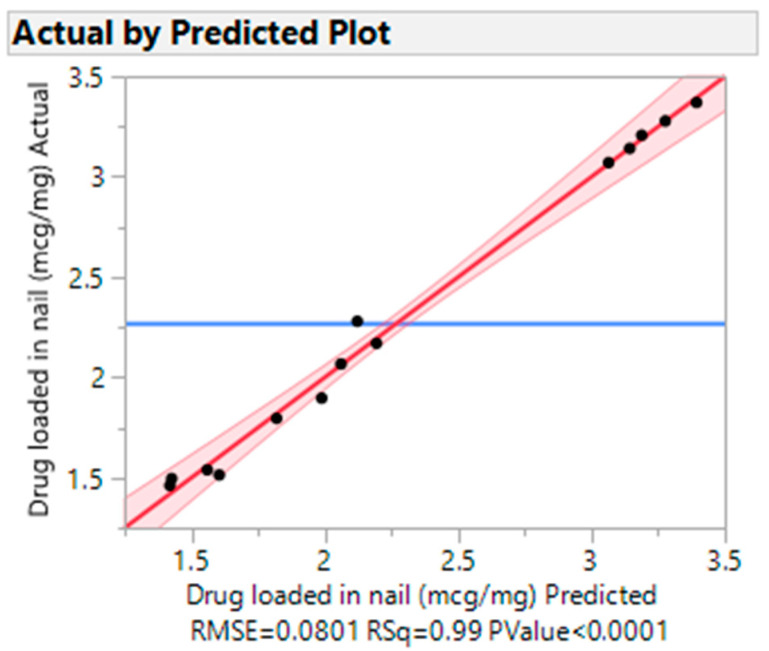
Actual vs. predicted plot for drug load.

A multiple regression model was employed to statistically assess the drug that was loaded into the nail and was presented in Figure 6. The prediction algorithm determined that there was a statistically significant drug load in the nail (R Square-0.99 and *p*-value 0.0001). The scaled estimates for drug loading through the nail were presented in Figure S3.

#### 3.4.3. Effects Summary Report

The results of the impact test report that was generated for each of the individual responses are summarized in Table 4. According to the effects test report for drug penetration in nails (*p*-0.0001), the voltage range has a significant impact on the model. The result of the test demonstrates that the permeation enhancer (PEG 400) also has a considerable impact on drug penetration (*p*-value, 0.0004). The impact test also indicates that the permeation enhancer and voltage range have a significant impact on the CQAs considered in the design. This indicates that models for drug penetration and drug loading in the nail were established, and the effects test showed that these models are statistically significant. 

The report provides the LogWorth values for each effect in the model. The controls in the report allow one to change the model’s impacts by adding or removing them. The model fit reports will automatically be updated thanks to the adjustments made to the impact summary report. The significant *p*-value and LogWorth values are shown in Table 5. The higher the value of LogWorth, the greater the potential of the variable’s influence on the quality characteristic. A comparison of values in Table 5 for the different variables indicates that the voltage range and penetration enhancer have greater significance than the solvent-to-co-solvent ratio.

Overall, the results here signify that the voltage is the most important factor affecting the transungual delivery of efinaconazole. With an increase in voltage, both drug diffusion through the membrane and deposition in the nail increase. Permeation enhancer seems to be the second important component that affects drug loading and penetration through the nail. In addition to the drug loading in the nail, an increase in the concentration (20–30%) of the permeation enhancer (PEG 400) considerably improved the efinaconazole penetration through the nail. Thirdly, the solvent: cosolvent effect is relatively less effective as compared to the other two variables. It was found that an increase in solvent (ethanol) decreases the drug diffusion through the nail and accumulation in the nail. However, the increased cosolvent (Labrasol^®^) concentration in the formulation enhances both permeation and drug load in the nail to a certain extent.

#### 3.4.4. Optimization Study Observations

**Figure 7:**
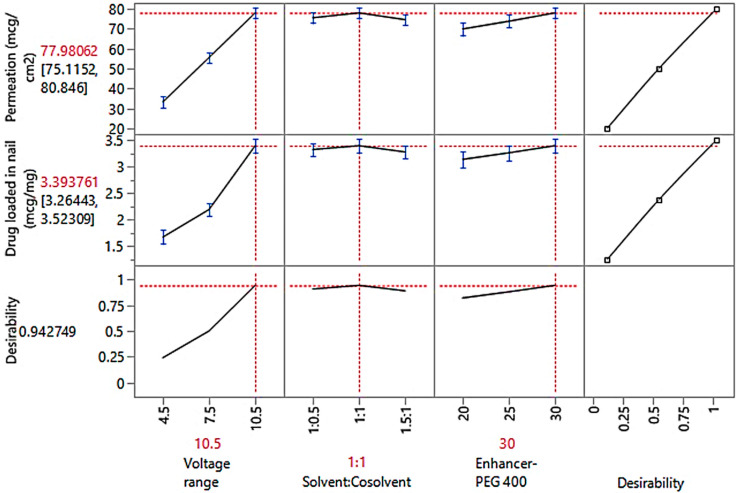
Prediction profiler.

The prediction profiler’s global desirability function was used to simultaneously optimize the transungual delivery of efinaconazole. A general sense of the variability achievable within the selected values is provided by the prediction profiler’s continual correlation between many factors and numerous responses. The high desirability value (0.9427) observed in Figure 7 indicates a strong correlation between the selected responses and independent variables, which is relevant to optimize the best formulation.

The optimized transungual iontophoretic delivery of efinaconazole comprised of specific voltage (10.5 V), solvent: cosolvent ratio (1:1), and enhancer-PEG 400 (30%) was prepared as per the prediction profiler to attain the CQAs considered in the design, and the same report was matched with the predicted responses. The experimental values and predicted values, as shown in Table 6, were quite similar. This demonstrated how well the optimization procedure could predict the qualities of the formulation.

The prediction profiler includes more built-in functionality. The “Maximize Desirability” function selects one set of factor settings that produces a predicted response that maximizes our desirability (as set in Response Limits). One should keep in mind that many factor-level combinations may enhance desirability. Figure 7 shows the optimum factor settings and associated response values after selecting the option of maximizing desire. The total desirability is approximately 94%.

### 3.5. Evaluation of Hydrogels

#### 3.5.1. Conductivity and Viscosity

Conductivity measurements provide a contextual indicator of the contribution of electrorepulsion to total iontophoretic flow. Table 3 shows the conductivity of prepared design batches. The observed values (3.51–4.93 mS) indicate the high conductivity of prepared batches and could be adequate for iontophoresis delivery, as described in the literature [66]. On the other hand, the conductivity value of saturated efinaconazole aqueous solution (3.5% *w*/*v*, pH 3) was moderately high (6.89 0.34 mS). The decrease in conductivity values of prepared batches could be attributed to the addition of ethanol (solvent), Labrasol^®^ (cosolvent), and PEG 400 (enhancer), as described in the literature [67]. In addition, the viscosity of prepared batches did not vary significantly between formulations (48–61 cPs), making it suitable for iontophoretic delivery, as reported earlier [68].

#### 3.5.2. FTIR Spectroscopy

**Figure 8:**
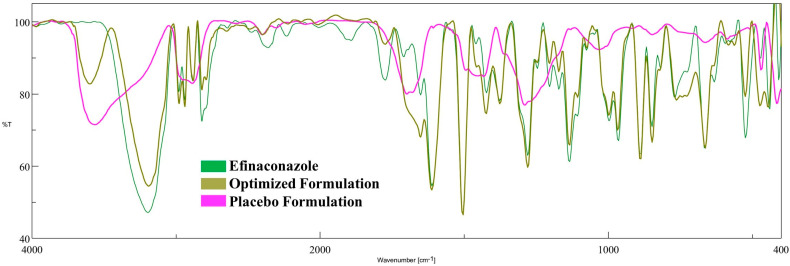
Fourier-transform infrared spectra of efinaconazole, placebo formulation, and optimized hydrogel.

Figure 8 represents the FTIR spectrum of efinaconazole, placebo, and optimized formulation. Pure drug shows O-H stretching bands at 3197.4 cm^−1^, aromatic C-H stretching bands at 2981.41 cm^−1^, aliphatic C-H stretching bands at 2938.98 cm^−1^, cyclic alkene C=C stretching bands at 1612.2 cm^−1^, alcohol O-H bending at 1423.21 cm^−1^, C-F fluoro compound at 1376.93 cm^−1^ and aromatic amine C-N stretching band at 1280.5 cm^−1^. The characteristic peaks for the drug and optimized formulation were evidenced with the theoretical estimation of the functional groups, suggesting no interaction of efinaconazole with the excipients used in the formulation. Indeed, the major characteristic peaks of the drug are not present in the placebo hydrogel.

#### 3.5.3. DSC Analysis

**Figure 9:**
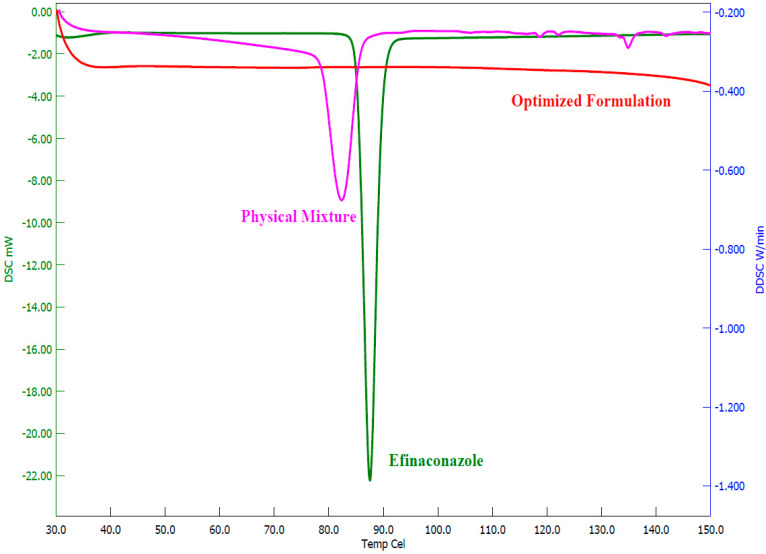
Differential scanning calorimetric patterns of efinaconazole, physical mixture, and optimized hydrogel.

The thermal analysis of the efinaconazole, physical mixture, and optimized hydrogel was recorded, and the respective thermograms were depicted in Figure 9. The pure efinaconazole displayed a typical endothermic peak at 87.5 °C, corresponding to the efinaconazole melting point. The drug melting curve was also visible in the physical mixture at 82.4 °C, although it was less intense and shifted to a somewhat lower temperature. The thermogram of the optimized formulation showed no drug characteristic peaks, which revealed the amorphization of the drug in the formulation.

#### 3.5.4. Field Emission SEM

**Figure 10:**
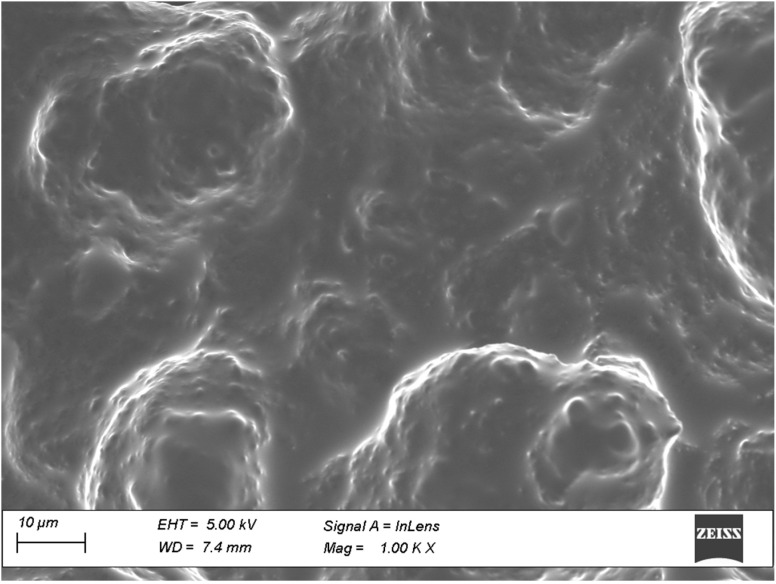
Field emission scanning electron microscope image of the optimized hydrogel.

Figure 10 shows the SEM image of optimized hydrogel. Visual inspection of the photograph reveals a gel-like consistency, complete dissolution of all formulation components, and the lack of any particles. The presence of PVP K30 in the formulation may be the cause of the film-like structure seen in the photograph. The existence of an irregular terrain might be caused by particulates produced due to the lyophilization of the product for SEM imaging, and it might not truly describe the actual product as stated in the literature [69].

**Figure 11:**
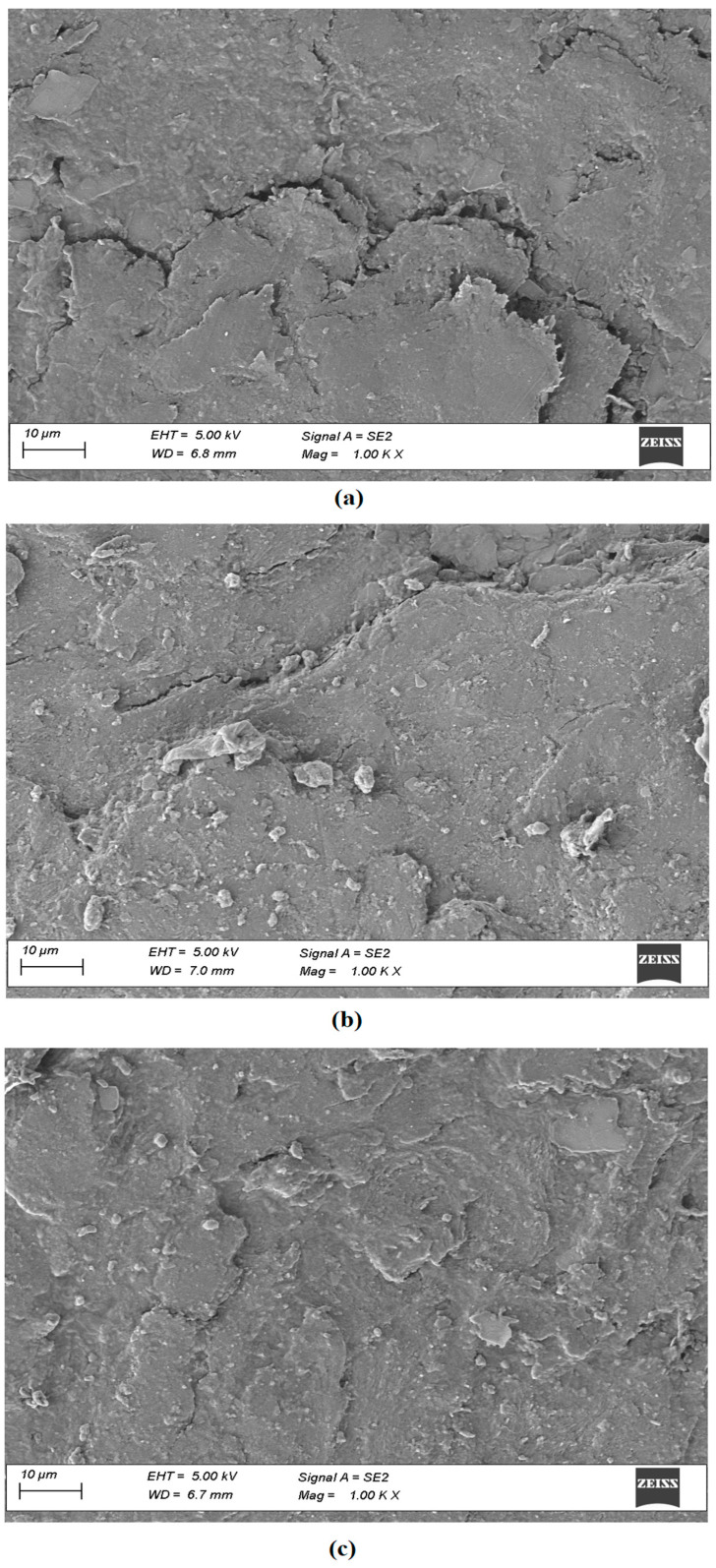
Scanning electron micrographs of (**a**) normal human nail plate, (**b**) nails treated with formulation, and (**c**) nails treated with formulation and iontophoresis.

The SEM micrographs in Figure 11 show the shape, roughness, and geometry of the upper surface of normal human nails (Figure 11a), nails treated with optimized formulation (Figure 11b), and nails treated with optimized formulation and iontophoresis (10.5 V) (Figure 11c). It is apparent from Figure 11 that the surface feature of the nail applied with formulation and iontophoresis is markedly different from the control nail (Figure 11a). The control nail shows a rough surface with few visible cracks, possibly due to cell-cell separation contributed by lack of hydration [70]. However, both treated nail groups have surfaces with closely packed cells that are relatively undisturbed, despite some visible apertures. These changes do not significantly affect the overall integrity of the nail structure. Furthermore, there is no noticeable difference between the nails treated with the formulation alone (Figure 11b) and those treated with formulation and iontophoresis (Figure 11c). In short, the findings indicate that the current technology has not altered the nail’s structure which is also in line with the usual finding in constant current iontophoresis [25].

### 3.6. Release of Efinaconazole from Loaded Nails

**Figure 12:**
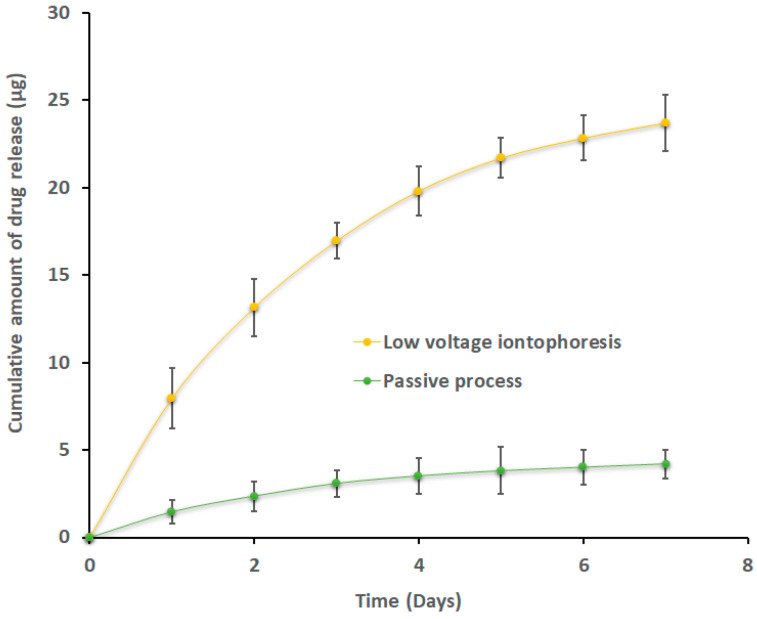
Release of efinaconazole from nails loaded by iontophoresis and passive process using optimized transungual iontophoretic delivery. The value represented is the mean ± SD (n = 6).

The design and development of topical nail products mainly aimed to provide drug entrapment in the nail membrane and subsequent release. This is because the drug depot that has built up in the nail plate eventually releases into the deeper regions of the nail bed, where the causative organism usually exists. Therefore, nail plates accumulated with efinaconazole were tested for ex vivo release, and the profiles are depicted in Figure 12. For nails loaded with efinaconazole via iontophoresis and passive process, two unique release profiles were seen. A greater release of efinaconazole (*p* < 0.0001) was seen in nails treated by iontophoresis when compared with the passive method. Additionally, the efinaconazole rate of release in the nails loaded by iontophoresis was significant on the first day, with ~13.15 µg, and declined later with time (~0.87 µg on the 7th day, Figure 12). The presence of a significantly greater amount of unbound efinaconazole in the nail could be directly linked to the cause of this higher rate of drug release in the early phase [33]. After the release of unbound efinaconazole, probably the remaining actives that are linked to keratin happen to release. At the end of the 7th day, the total amount of efinaconazole released was 23.69 ± 0.87 µg (or ~66% of the total efinaconazole accumulated in the nail by optimized transungual iontophoretic delivery). It is worthwhile to note that efinaconazole has a low affinity with nail keratin, which enables them to have a drug free for release/permeation [71]. In contrast, the efinaconazole release from nails loaded by a passive process (without iontophoresis) seems to be low and slow. In conclusion, the results presented here showed that efinaconazole accumulation in the membrane caused by iontophoresis results in increased efinaconazole release, which may be beneficial due to steady release of efinaconazole to the nail bed might eradicate organisms and prevent a recurrence.

### 3.7. Antifungal Study

**Figure 13:**
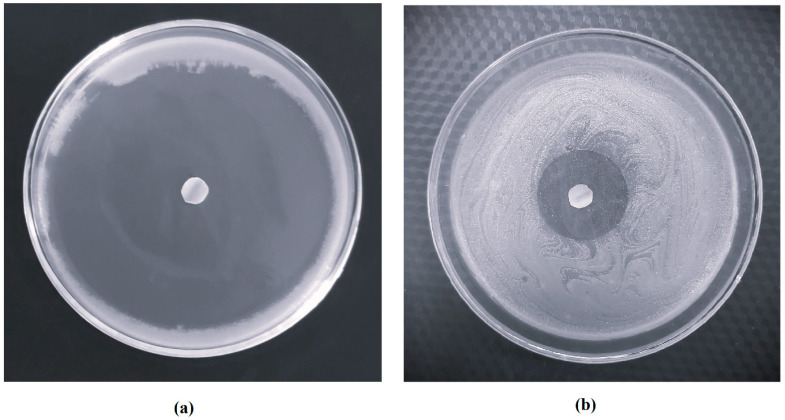
Comparison of the zone of inhibition of nails loaded by (**a**) iontophoresis (10.5 V) and (**b**) passive (0 V) process using optimized hydrogel formulation.

The pharmacodynamic activity of efinaconazole loaded in the nail plate is of utmost importance to provide good clinical outcomes. In the case of onychomycosis, the fungi reside in the nail membrane as well as in the nail bed; hence, the efficacy of the transungual delivery depends on the amount of drug diffused through the nail and as well as that has passed through the nail membrane [39]. Hence, to assess the antifungal efficiency of efinaconazole, the inside of the nail matrix was checked against fungal strains of *Trichophyton mentagrophyte.* The method used here evaluates how much efinaconazole is released from nails, spreads into the media, and prevents the growth of microorganisms, which suggests an antifungal activity. The zone of inhibition that appeared on day 4 for both treatments is presented in Figure 13. It can be seen that the zone of inhibition is very prominent with iontophoretic treatment (83.7 ± 3.2 mm, ~300% higher) when compared with the passive process (26.5 ± 2.1 mm). The observed enhancement in fungicidal activity could be linked to the greater efinaconazole loading that occurs after the iontophoretic technique (3.24 ± 0.35 µg/mg) when compared to the normal process (1.29 ± 0.24 µg/mg). The release data observed in Figure 12, which was much faster with iontophoretic loaded nails, is also supported by these findings. On the other hand, a low antimicrobial effect was noticed by the smaller inhibition zone seen in nails loaded using the passive approach. In summary, the findings imply that efinaconazole establishes a depot in the nail, causing a steady release of the drug over time with an impact on both the nail plate and bed. Thus, constant voltage iontophoresis seems endowed with more effective management of onychomycosis and potentially assures the complete eradication of pathogenic dermatophytes.

### 3.8. Formulation Stability

Efinaconazole-containing formulations have a history of discoloring within a short span [39]. However, current formulations comprised a specific percentage (0.01% *w*/*v*) of antioxidants, namely, EDTA and BHT did not show any discoloration. The hydrogel was found homogeneous, with no alteration in color, aggregate, or sedimentation. In addition, no variation in the drug loading (7.33 ± 0.28%), pH (3.47 ± 0.24), or viscosity (51.99 ± 2.60 cP at 50 rpm) was observed after the stipulated study period. In conclusion, this study confirms that the prepared hydrogel possesses adequate stability when stored for 3 months at controlled stability conditions.

## 4. Conclusions

A systematic investigation was performed to assess the feasibility of constant voltage iontophoresis to improve the nail permeation of efinaconazole. Initial studies were done to select pharmaceutically acceptable solvents, cosolvents, and enhancers to develop a hydrogel composition. The evaluation of prototype formulations (E1–E7) signified a moderate effect on drug delivery when the solvent-to-cosolvent ratio in the hydrogel composition was changed. Optimized transungual iontophoretic delivery results indicate a significant influence of applied voltage and concentration of permeation enhancer on the critical quality attributes. Indeed, the increase in voltage or enhancer concentration significantly improved the efinaconazole diffusion and accumulation in the nail. The DSC and FTIR data validated the amorphous state of efinaconazole and the absence of interaction between the efinaconazole and other excipients, respectively, in the optimized hydrogel. A higher efinaconazole release (*p* < 0.0001) for one week observed from nails loaded by iontophoresis might decrease the interval of topical application. Extremely high inhibition observed in nails loaded by iontophoresis (~300% higher than the passive process) signifies that the efinaconazole depot formed in the nail plate is therapeutically active and can exert fungicidal activity. In conclusion, the proposed technique could be a patient-compliant non-invasive approach for delivering antimycotic agents topically and would improve the treatment of onychomycosis.

## Figures and Tables

**Table 1:** Composition of primary formulations (E1–E7) containing efinaconazole.

Ingredients (% *w*/*w*)	Batch Code						
	E1	E2	E3	E4	E5	E6	E7
Efinaconazole	7.5	7.5	7.5	7.5	7.5	7.5	7.5
Ethyl alcohol	5	10	15	20	15	25	25
Labrasol^®^	5	5	5	5	10	10	15
Tween 80	5	5	5	5	5	5	5
Polyethylene glycol 400	25	25	25	25	25	25	25
Polyvinylpyrrolidone K30	1	1	1	1	1	1	1
Disodium ethylenediaminetetraacetic acid	0.01	0.01	0.01	0.01	0.01	0.01	0.01
Butylated hydroxytoluene	0.01	0.01	0.01	0.01	0.01	0.01	0.01
Water (up to)	100	100	100	100	100	100	100

**Table 2:** Physicochemical characteristics of primary formulations (E1–E7).

Parameters	Batch Code						
	E1	E2	E3	E4	E5	E6	E7
pH	3.16 ± 0.12	3.05 ± 0.08	3.11 ± 0.05	3.20 ± 0.14	3.15 ± 0.06	3.08 ± 0.13	3.12 ± 0.14
Loading efficacy (%)	98.20 ± 1.97	97.02 ± 2.31	98.44 ± 1.55	98.30 ± 1.55	97.84 ± 2.11	98.61 ± 1.08	97.92 ± 1.95
Drug loading (%)	7.45 ± 0.22	7.40 ± 0.16	7.48 ± 0.18	7.52 ± 0.05	7.41 ± 0.21	7.45 ± 0.16	7.47 ± 0.15
Viscosity (cP) at 50 rpm	56.92 ± 1.85	59.00 ± 2.31	57.76 ± 2.22	61.11 ± 2.61	68.42 ± 2.37	67.22 ± 2.01	72.87 ± 3.54

**Table 3:** Composition of design batches as per the custom design and characterization report.

Formulation	Voltage Range (V)	Solvent:Cosolvent	Enhancer-PEG 400 (%)	Conductivity (mS)	Permeation (µg/cm^2^)	Drug Loaded in the Nail (µg/mg)
F8	4.5	1:0.5	30	4.16 ± 0.13	28.65 ± 3.05	1.51 ± 0.16
F10	4.5	1:1	20	4.66 ± 0.11	24.80 ± 2.64	1.46 ± 0.26
F15	4.5	1.5:1	25	4.02 ± 0.06	27.78 ± 3.56	1.50 ± 0.17
F12	4.5	1.5:1	30	3.51 ± 0.10	31.03 ± 2.92	1.54 ± 0.15
F7	7.5	1:0.5	25	4.52 ± 0.12	47.21 ± 2.89	1.90 ± 0.24
F4	7.5	1:0.5	30	4.16 ± 0.13	55.69 ± 4.05	2.28 ± 0.31
F3	7.5	1:1	25	4.28 ± 0.08	51.98 ± 3.84	2.07 ± 0.25
F9	7.5	1:1	30	3.82 ± 0.05	55.03 ± 4.63	2.17 ± 0.29
F1	7.5	1.5:1	20	4.38 ± 0.15	43.94 ± 3.61	1.80 ± 0.26
F11	7.5	1.5:1	20	4.38 ± 0.15	43.94 ± 3.60	1.80 ± 0.21
F2	10.5	1:0.5	20	4.93 ± 0.09	68.56 ± 5.36	3.07 ± 0.31
F6	10.5	1:0.5	25	4.52 ± 0.12	71.94 ± 5.20	3.21 ± 0.33
F14	10.5	1:1	30	3.82 ± 0.05	78.66 ± 5.66	3.37 ± 0.35
F13	10.5	1.5:1	25	4.02 ± 0.06	69.38 ± 5.08	3.14 ± 0.11
F5	10.5	1.5:1	30	3.51 ± 0.10	73.39 ± 3.96	3.28 ± 0.31

**Table 4:** Effect test report for efinaconazole diffusion and accumulation inside the nail.

Source	Efinaconazole Diffusion					Efinaconazole Accumulation inside the Nail				
	Nparm	DF	Sum of Squares	F Ratio	Prob > F	Nparm	DF	Sum of Squares	F Ratio	Prob > F
Voltage range	2	2	4316.5128	685.9742	<0.0001	2	2	7.0468285	549.7144	<0.0001
Solvent: cosolvent	2	2	26.6675	4.2380	0.0556	2	2	0.0315546	2.4615	0.1469
Enhancer-PEG 400	2	2	151.9646	24.1500	0.0004	2	2	0.1549844	12.0901	0.0038

**Table 5:** Effects summary for the whole model.

Source	LogWorth	*p* Value
Voltage range	8.947	0.00000
Enhancer-PEG 400	3.390	0.00041
Solvent:cosolvent	1.255	0.05559

**Table 6:** Predicted and experimental values obtained for optimized transungual iontophoretic delivery.

Optimized Transungual Iontophoretic Delivery					
Drug Permeation (µg/cm^2^)		% Difference	Drug Loading (µg/mg)		% Difference
Predicted	Observed		Predicted	Observed	
77.98	78.59	0.782	3.39	3.2	−2.06

% Difference = ((Observed − Predicted)/(Predicted)) × 100.

## Data Availability

The data presented in this study are contained within the article.

## Supplementary Materials

The following supporting information can be downloaded at: https://www.mdpi.com/article/10.3390/pharmaceutics15051422/s1, Figure S1. Representative HPLC chromatogram of efinaconazole; Figure S2. Scaled estimates for drug permeation; Figure S3. Scaled estimates for drug loading.

## References

[1] Ghannoum M., Isham N. (2014). Fungal nail infections (onychomycosis): A never-ending story?. PLoS Pathog..

[2] Kushwaha A.S., Sharma P., Shivakumar H.N., Rappleye C., Zukiwski A., Proniuk S., Murthy S.N. (2017). Trans-ungual Delivery of AR-12, a Novel Antifungal Drug. AAPS PharmSciTech..

[3] Elewski B.E., Tosti A. (2015). Risk Factors and Comorbidities for Onychomycosis: Implications for Treatment with Topical Therapy. J. Clin. Aesthetic Dermatol..

[4] Nair A.B., Vaka S.R., Murthy S.N. (2011). Transungual delivery of terbinafine by iontophoresis in onychomycotic nails. Drug Dev. Ind. Pharm..

[5] Kawa N., Lee K.C., Anderson R.R., Garibyan L. (2019). ONYCHOMYCOSIS: A Review of New and Emerging Topical and Device-based Treatments. J. Clin. Aesthetic Dermatol..

[6] Elkeeb R., Hui X., Murthy N., Maibach H.I. (2014). Emerging topical onychomycosis therapies-quo vadis?. Expert Opin. Emerg. Drugs.

[7] Dias M.F., Quaresma-Santos M.V., Bernardes-Filho F., Amorim A.G., Schechtman R.C., Azulay D.R. (2013). Update on therapy for superficial mycoses: Review article part I. An. Bras. Dermatol..

[8] Gupta A.K., Stec N., Summerbell R.C., Shear N.H., Piguet V., Tosti A., Piraccini B.M. (2020). Onychomycosis: A review. J. Eur. Acad. Dermatol. Venereol. JEADV.

[9] Del Rosso J.Q. (2014). The role of topical antifungal therapy for onychomycosis and the emergence of newer agents. J. Clin. Aesthetic Dermatol..

[10] Ghannoum M., Isham N., Catalano V. (2014). A second look at efficacy criteria for onychomycosis: Clinical and mycological cure. Br. J. Dermatol..

[11] Akhtar N., Sharma H., Pathak K. (2016). Onychomycosis: Potential of Nail Lacquers in Transungual Delivery of Antifungals. Scientifica.

[12] Shanbhag P.P., Jani U. (2017). Drug delivery through nails: Present and future. New Horiz. Transl. Med..

[13] Murdan S. (2008). Enhancing the nail permeability of topically applied drugs. Expert Opin. Drug Deliv..

[14] Nair A.B., Sammeta S.M., Vaka S.R., Narasimha Murthy S. (2009). A study on the effect of inorganic salts in transungual drug delivery of terbinafine. J. Pharm. Pharmacol..

[15] Nair A.B., Sammeta S.M., Kim H.D., Chakraborty B., Friden P.M., Murthy S.N. (2009). Alteration of the diffusional barrier property of the nail leads to greater terbinafine drug loading and permeation. Int. J. Pharm..

[16] Murthy S.N., Vaka S.R.K., Sammeta S.M., Nair A.B. (2009). TranScreen-N™: Method for rapid screening of trans-ungual drug delivery enhancers. J. Pharm. Sci..

[17] Saner M.V., Kulkarni A.D., Pardeshi C.V. (2014). Insights into drug delivery across the nail plate barrier. J. Drug Target..

[18] Šveikauskaitė I., Pockevičius A., Briedis V. (2019). Potential of Chemical and Physical Enhancers for Transungual Delivery of Amorolfine Hydrochloride. Materials.

[19] Shivakumar H.N., Juluri A., Desai B.G., Murthy S.N. (2012). Ungual and transungual drug delivery. Drug Dev. Ind. Pharm..

[20] Albarahmieh E., AbuAmmouneh L., Kaddoura Z., AbuHantash F., Alkhalidi B.A., Al-Halhouli A. (2019). Fabrication of Dissolvable Microneedle Patches Using an Innovative Laser-Cut Mould Design to Shortlist Potentially Transungual Delivery Systems: In Vitro Evaluation. AAPS PharmSciTech.

[21] Elsherif N.I., Shamma R.N., Abdelbary G. (2017). Terbinafine Hydrochloride Trans-ungual Delivery via Nanovesicular Systems: In Vitro Characterization and Ex Vivo Evaluation. AAPS PharmSciTech.

[22] El-sherif N.I., Shamma R.N., Abdelbary G. (2018). In-situ gels and nail lacquers as potential delivery systems for treatment of onychomycosis. A comparative study. J. Drug Deliv. Sci. Technol..

[23] Tanrıverdi S.T., Özer Ö. (2013). Novel topical formulations of Terbinafine-HCl for treatment of onychomycosis. Eur. J. Pharm. Sci. Off. J. Eur. Fed. Pharm. Sci..

[24] Bhuptani R.S., Deshpande K.M., Patravale V.B. (2016). Transungual permeation: Current insights. Drug Deliv. Transl. Res..

[25] Cordoba Díaz D., Losa Iglesias M.E., Becerro de Bengoa Vallejo R., Cordoba Diaz M. (2019). Transungual Delivery of Ciclopirox Is Increased 3⁻4-Fold by Mechanical Fenestration of Human Nail Plate in an In Vitro Model. Pharmaceutics.

[26] Hao J., Smith K.A., Li S.K. (2009). Iontophoretically enhanced ciclopirox delivery into and across human nail plate. J. Pharm. Sci..

[27] Monti D., Mazzantini D., Tampucci S., Vecchione A., Celandroni F., Burgalassi S., Ghelardi E. (2019). Ciclopirox and Efinaconazole Transungual Permeation, Antifungal Activity, and Proficiency To Induce Resistance in Trichophyton rubrum. Antimicrob. Agents Chemother..

[28] Nair A.B., Kim H.D., Davis S.P., Etheredge R., Barsness M., Friden P.M., Murthy S.N. (2009). An ex vivo toe model used to assess applicators for the iontophoretic ungual delivery of terbinafine. Pharm. Res..

[29] Uchida N., Yanagi M., Hamada H. (2021). Physical Enhancement? Nanocarrier? Current Progress in Transdermal Drug Delivery. Nanomaterials.

[30] Mahmud M., Rahman A., Salem K.S., Bari M.L., Qiu H. (2022). Architecting Ultrathin Graphitic C(3)N(4) Nanosheets Incorporated PVA/Gelatin Bionanocomposite for Potential Biomedical Application: Effect on Drug Delivery, Release Kinetics, and Antibacterial Activity. ACS Appl. Bio. Mater..

[31] Suh T.C., Twiddy J., Mahmood N., Ali K.M., Lubna M.M., Bradford P.D., Daniele M.A., Gluck J.M. (2022). Electrospun Carbon Nanotube-Based Scaffolds Exhibit High Conductivity and Cytocompatibility for Tissue Engineering Applications. ACS Omega.

[32] Nair A.B., Kim H.D., Chakraborty B., Singh J., Zaman M., Gupta A., Friden P.M., Murthy S.N. (2009). Ungual and trans-ungual iontophoretic delivery of terbinafine for the treatment of onychomycosis. J. Pharm. Sci..

[33] Nair A.B., Vaka S.R., Sammeta S.M., Kim H.D., Friden P.M., Chakraborty B., Murthy S.N. (2009). Trans-ungual iontophoretic delivery of terbinafine. J. Pharm. Sci..

[34] Nair A.B., Al-Dhubiab B.E., Shah J., Gorain B., Jacob S., Attimarad M., Sreeharsha N., Venugopala K.N., Morsy M.A. (2021). Constant Voltage Iontophoresis Technique to Deliver Terbinafine via Transungual Delivery System: Formulation Optimization Using Box–Behnken Design and In Vitro Evaluation. Pharmaceutics.

[35] Bhatt V., Pillai R. (2015). Efinaconazole topical solution, 10%: Formulation development program of a new topical treatment of toenail onychomycosis. J. Pharm. Sci..

[36] Gupta A.K., Venkataraman M., Renaud H.J., Summerbell R., Shear N.H., Piguet V. (2021). A Paradigm Shift in the Treatment and Management of Onychomycosis. Skin Appendage Disord..

[37] Hur M.S., Park M., Jung W.H., Lee Y.W. (2019). Evaluation of drug susceptibility test for Efinaconazole compared with conventional antifungal agents. Mycoses.

[38] Kircik L.H. (2014). Enhancing transungual delivery and spreading of efinaconazole under the nail plate through a unique formulation approach. J. Drugs Dermatol. JDD.

[39] Lee B.C., Pangeni R., Na J., Koo K.T., Park J.W. (2019). Preparation and in vivo evaluation of a highly skin- and nail-permeable efinaconazole topical formulation for enhanced treatment of onychomycosis. Drug Deliv..

[40] Park J.S., Kim J.S., Ho M.J., Park D.W., Kim E.A., Choi Y.S., Jang S.W., Kang M.J. (2021). Effect of Penetration Enhancers on Toenail Delivery of Efinaconazole from Hydroalcoholic Preparations. Molecules.

[41] Aggarwal R., Targhotra M., Sahoo P., Chauhan M.K. (2020). Efinaconazole nail lacquer for the transungual drug delivery: Formulation, optimization, characterization and in vitro evaluation. J. Drug Deliv. Sci. Technol..

[42] Almuqbil R.M., Sreeharsha N., Nair A.B. (2022). Formulation-by-Design of Efinaconazole Spanlastic Nanovesicles for Transungual Delivery Using Statistical Risk Management and Multivariate Analytical Techniques. Pharmaceutics.

[43] Anroop B., Ghosh B., Parcha V., Kumar A., Khanam J. (2005). Synthesis and comparative skin permeability of atenolol and propranolol esters. J. Drug Deliv. Sci. Technol..

[44] Shah H., Nair A.B., Shah J., Bharadia P., Al-Dhubiab B.E. (2019). Proniosomal gel for transdermal delivery of lornoxicam: Optimization using factorial design and in vivo evaluation in rats. DARU J. Pharm. Sci..

[45] Vörös-Horváth B., Das S., Salem A., Nagy S., Böszörményi A., Kőszegi T., Pál S., Széchenyi A. (2020). Formulation of Tioconazole and Melaleuca alternifolia Essential Oil Pickering Emulsions for Onychomycosis Topical Treatment. Molecules.

[46] Mady O.Y., Al-Madboly L.A., Donia A.A. (2020). Preparation, and Assessment of Antidermatophyte Activity of Miconazole-Urea Water-Soluble Film. Front. Microbiol..

[47] Guideline I.H.T. (2003). Stability testing of new drug substances and products. Q1A (R2) Curr. Step.

[48] Wang Y., Zeng L., Song W., Liu J. (2022). Influencing factors and drug application of iontophoresis in transdermal drug delivery: An overview of recent progress. Drug Deliv. Transl. Res..

[49] Anroop B., Ghosh B., Parcha V., Khanam J. (2009). Transdermal delivery of atenolol: Effect of prodrugs and iontophoresis. Curr. Drug Deliv..

[50] Nair A., Vyas H., Shah J., Kumar A. (2011). Effect of permeation enhancers on the iontophoretic transport of metoprolol tartrate and the drug retention in skin. Drug Deliv..

[51] Nair A., Reddy C., Jacob S. (2009). Delivery of a classical antihypertensive agent through the skin by chemical enhancers and iontophoresis. Skin Res. Technol..

[52] Kreutz T., de Matos S.P., Koester L.S. (2019). Recent Patents on Permeation Enhancers for Drug Delivery Through Nails. Recent Pat. Drug Deliv. Formul..

[53] Nair A.B., Chakraborty B., Murthy S.N. (2010). Effect of polyethylene glycols on the trans-ungual delivery of terbinafine. Curr. Drug Deliv..

[54] Myoung Y., Choi H.K. (2003). Permeation of ciclopirox across porcine hoof membrane: Effect of pressure sensitive adhesives and vehicles. Eur. J. Pharm. Sci. Off. J. Eur. Fed. Pharm. Sci..

[55] Cutrín-Gómez E., Anguiano-Igea S., Delgado-Charro M.B., Gómez-Amoza J.L., Otero-Espinar F.J. (2018). Effect of Penetration Enhancers on Drug Nail Permeability from Cyclodextrin/Poloxamer-Soluble Polypseudorotaxane-Based Nail Lacquers. Pharmaceutics.

[56] Alqahtani A., Raut B., Khan S., Mohamed J.M.M., Fatease A.A., Alqahtani T., Alamri A., Ahmad F., Krishnaraju V. (2022). The Unique Carboxymethyl Fenugreek Gum Gel Loaded Itraconazole Self-Emulsifying Nanovesicles for Topical Onychomycosis Treatment. Polymers.

[57] Jacob S., Nair A.B., Patel V., Shah J. (2020). 3D Printing Technologies: Recent Development and Emerging Applications in Various Drug Delivery Systems. AAPS PharmSciTech.

[58] Kurakula M., Rao G. (2020). Pharmaceutical assessment of polyvinylpyrrolidone (PVP): As excipient from conventional to controlled delivery systems with a spotlight on COVID-19 inhibition. J. Drug Deliv. Sci. Technol..

[59] Pal P., Thakur R.S., Ray S., Mazumder B. (2015). Design and development of a safer non-invasive transungual drug delivery system for topical treatment of onychomycosis. Drug Dev. Ind. Pharm..

[60] Vejnovic I., Simmler L., Betz G. (2010). Investigation of different formulations for drug delivery through the nail plate. Int. J. Pharm..

[61] Smith K.A., Hao J., Li S.K. (2011). Effects of organic solvents on the barrier properties of human nail. J. Pharm. Sci..

[62] Agrawal V., Patel R., Patel M., Thanki K., Mishra S. (2021). Design and evaluation of microemulsion-based efinaconazole formulations for targeted treatment of onychomycosis through transungual route: Ex vivo and nail clipping studies. Colloids Surf. B Biointerfaces.

[63] McCartney F., Jannin V., Chevrier S., Boulghobra H., Hristov D.R., Ritter N., Miolane C., Chavant Y., Demarne F., Brayden D.J. (2019). Labrasol® is an efficacious intestinal permeation enhancer across rat intestine: Ex vivo and in vivo rat studies. J. Control. Release Off. J. Control. Release Soc..

[64] Liu Z., Zhang X., Li J., Liu R., Shu L., Jin J. (2009). Effects of Labrasol on the corneal drug delivery of baicalin. Drug Deliv..

[65] Panapisal V., Charoensri S., Tantituvanont A. (2012). Formulation of microemulsion systems for dermal delivery of silymarin. AAPS PharmSciTech.

[66] Zhou Y., Jia X., Pang D., Jiang S., Zhu M., Lu G., Tian Y., Wang C., Chao D., Wallace G. (2023). An integrated Mg battery-powered iontophoresis patch for efficient and controllable transdermal drug delivery. Nat. Commun..

[67] Chen K., Puri V., Michniak-Kohn B. (2021). Iontophoresis to Overcome the Challenge of Nail Permeation: Considerations and Optimizations for Successful Ungual Drug Delivery. The AAPS J..

[68] Teaima M.H., Mohamed M.A.A., Abd El Rehem R.T., Tayel S.A., El-Nabarawi M.A., Fouad S.A. (2021). Enhanced Transdermal Delivery of Bisoprolol Hemifumarate via Combined Effect of Iontophoresis and Chemical Enhancers: Ex Vivo Permeation/In Vivo Pharmacokinetic Studies. Pharmaceutics.

[69] Town A.R., Taylor J., Dawson K., Niezabitowska E., Elbaz N.M., Corker A., Garcia-Tuñón E., McDonald T.O. (2019). Tuning HIV drug release from a nanogel-based in situ forming implant by changing nanogel size. J. Mater. Chem. B.

[70] Baraldi A., Jones S.A., Guesné S., Traynor M.J., McAuley W.J., Brown M.B., Murdan S. (2015). Human nail plate modifications induced by onychomycosis: Implications for topical therapy. Pharm. Res..

[71] Sugiura K., Sugimoto N., Hosaka S., Katafuchi-Nagashima M., Arakawa Y., Tatsumi Y., Jo Siu W., Pillai R. (2014). The low keratin affinity of efinaconazole contributes to its nail penetration and fungicidal activity in topical onychomycosis treatment. Antimicrob. Agents Chemother..

