# Supplementary material for: Design, Development, and Evaluation of Constant Voltage Iontophoresis for the Transungual Delivery of Efinaconazole
Source: Pharmaceutics. 2023 May 6;15(5):1422. doi: 10.3390/pharmaceutics15051422 (PMC10223599; doi:10.3390/pharmaceutics15051422)
Supplement: Supplementary file 1 [file pharmaceutics-15-01422-s001.zip › pharmaceutics-2192985-supplementary.pdf]

pharmaceutics

MDPI

Design, Development, and Evaluation of Constant Voltage Iontophoresis for the Transungual Delivery of Efinaconazole

Anroop B. Nair, Bandar Aldhubiab, Jigar Shah, Shery Jacob, Mahesh Attimarad, Nagaraja Sreeharsha, Katharigatta N. Venugopala, Alex Joseph and Mohamed A. Morsy

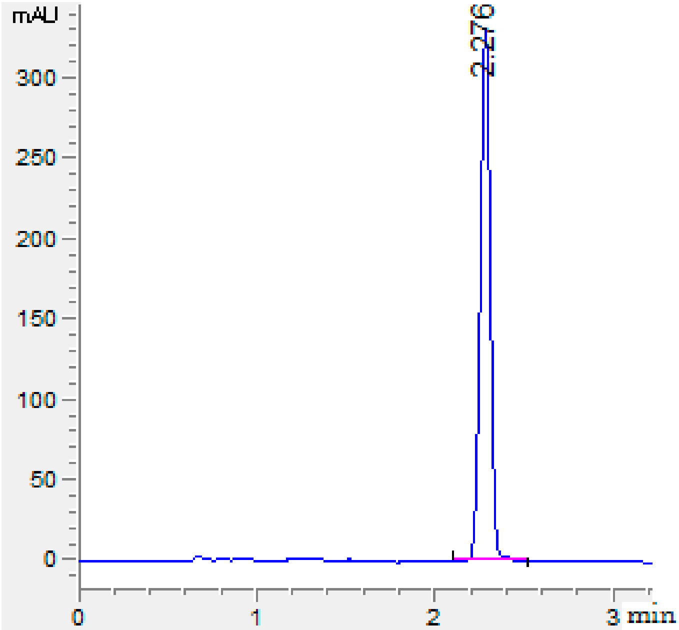

Figure S1. Representative HPLC chromatogram of efinaconazole.

Scaled Estimates

Nominal factors expanded to all levels

Term	Scaled Estimate		Std Error	t Ratio	Prob>|t|
Intercept	49.66827		0.482759	102.88	<.0001*
Voltage range[4.5]	-22.26589		0.703737	-31.64	<.0001*
Voltage range[7.5]	-0.038118		0.638631	-0.06	0.9539
Voltage range[10.5]	22.304007		0.661046	33.74	<.0001*
Solvent:Cosolvent[1:0.5]	-0.509859		0.661046	-0.77	0.4627
Solvent:Cosolvent[1:1]	1.9378624		0.703737	2.75	0.0249*
Solvent:Cosolvent[1.5:1]	-1.428003		0.638631	-2.24	0.0558
Enhancer- PEG400[20]	-3.993007		0.703737	-5.67	0.0005*
Enhancer- PEG400[25]	-0.077471		0.661046	-0.12	0.9096
Enhancer- PEG400[30]	4.0704782		0.638631	6.37	0.0002*

Figure S2. Scaled estimates for drug permeation. *significant

Scaled Estimates

Nominal factors expanded to all levels

Term	Scaled Estimate	Std Error	t Ratio	Prob>|t|
Intercept	2.2282625	0.021789	102.26	<.0001*
Voltage range[4.5]	-0.744747	0.031763	-23.45	<.0001*
Voltage range[7.5]	-0.227368	0.028825	-7.89	<.0001*
Voltage range[10.5]	0.9721154	0.029836	32.58	<.0001*
Solvent:Cosolvent[1:0.5]	-0.009115	0.029836	-0.31	0.7678
Solvent:Cosolvent[1:1]	0.0631313	0.031763	1.99	0.0821
Solvent:Cosolvent[1.5:1]	-0.054017	0.028825	-1.87	0.0978
Enhancer- PEG400[20]	-0.127101	0.031763	-4.00	0.0039*
Enhancer- PEG400[25]	-0.003151	0.029836	-0.11	0.9185
Enhancer- PEG400[30]	0.1302518	0.028825	4.52	0.0020*

Figure S3. Scaled estimates for drug loading. *significant
